# Editorial: Exploring structural variants in plant pangenomics: innovations and applications

**DOI:** 10.3389/fpls.2025.1700222

**Published:** 2025-10-08

**Authors:** Jinglong Wang, Qilong Zhou, Yunfei Liu, Risheng Li, Lan Lan, Junliang Zhao, Zefeng Yang, Yong Jia, Haifei Hu

**Affiliations:** ^1^ Institute of Pratacultural Science, Xizang Academy of Agricultural and Animal Husbandry Sciences, Lhasa, China; ^2^ Key Laboratory of Genetics and Breeding of High Quality Rice in Southern China, Ministry of Agriculture and Rural Affairs, Guangdong Key Laboratory of Rice Science and Technology, Guangdong Rice Engineering Laboratory, Rice Research Institute, Guangdong Academy of Agricultural Sciences, Guangzhou, China; ^3^ Western Crop Genetics Alliance, College of Science, Health, Engineering and Education, Murdoch University, Murdoch, WA, Australia; ^4^ Jiangsu Key Laboratory of Crop Genetics and Physiology, Jiangsu Key Laboratory of Crop Cultivation and Physiology, Agricultural College of Yangzhou University, Yangzhou, Jiangsu, China

**Keywords:** pangenome, structural variants, genetic diversity, plant breeding, biotic and abiotic response

Structural variants (SVs), which include large insertions, deletions, duplications, inversions, and translocations, have emerged as pivotal drivers of genomic diversity in plants. Unlike single-nucleotide changes, SVs can drastically alter gene content and genome architecture, thereby influencing phenotypic traits and adaptive potential (Hu et al., 2024a). The advent of plant pangenomes, which capture the full spectrum of genetic variation across multiple accessions of a species, has revolutionized our understanding of how SVs contribute to evolution and crop improvement (Hu et al., 2024b). By moving beyond a single reference genome, pangenomic analyses reveal missing genes and alleles, uncovering SV-linked traits that traditional single-reference genome-based approaches often overlook (Tong et al., 2025; Wang et al., 2023). This Research Topic focuses on these advances, highlighting studies that leverage pangenome frameworks to elucidate the roles of SVs in genetic diversity, environmental adaptation, and agronomic traits. We synthesize the findings of the contributions in this Research Topic and place them within the broader context of recent large-scale comparative studies in crops such as rice and barley, which also demonstrate the transformative impact of SV analysis on plant genomics.

## Pangenome perspectives on gene presence/absence variation in stress adaptation

Multiple studies included in this Research Topic have examined how PAV of gene families across plant pangenomes can illuminate stress tolerance mechanisms. Man et al. analyzed the auxin response factor (ARF) gene family across a 26-genome maize pangenome (Hufford et al., 2021), revealing substantial variations in gene content and stress-induced expression. Among these, *ARF4* consistently exhibited upregulation under both drought and salt stress, underscoring its role as a key regulator of abiotic stress responses. Similarly, Fan et al. built an 18-genome *Brassica napus* pangenome and identified 353 *R2R3-MYB* gene clusters, revealing extensive gene PAV and stress-responsive expression variation that highlights candidate regulators of abiotic stress adaptation. Two contributions focused specifically on PAV variations across different cultivars and explored its functional implications. Wang et al. analyzed the CBF gene family, which is associated with cold stress responses, in five yellowhorn cultivars. The authors identified eight dispensable and unique gene members. Notably, a cultivar-specific gene, *Xg11_CBF11*, enhanced cold tolerance when expressed in *Arabidopsis*. Similarly, Ma et al. identified 149 *PSKR* genes in allohexaploid wheat, unevenly distributed across 21 chromosomes. Expression analyses revealed that many of these genes are stress- and hormone-responsive, providing a resource of candidate *PSKRs* for improving wheat resilience. Across these studies, a unifying theme is that pangenome-based analyses of plant species reveal gene PAV that can be crucial for environmental adaptation and agronomic traits. The ability to link such SV-driven gene PAV to stress tolerance underscores the practical value of pangenomics for breeding improvement.

## Evolutionary and comparative genomics of structural variation

Expanding to evolutionary scales, multi-genome analyses demonstrate that SVs repeatedly remodel gene families and defense repertoires across plant lineages. In Rosaceae, for example, Yang et al. showed that the sorbitol-6-phosphate dehydrogenase (*S6PDH*) family originated from ancestral duplications and subsequently diversified through whole-genome duplication in Maleae and transpositions in peach, producing gene clusters with distinct metabolic roles. Similarly, Fan et al. revealed contrasting strategies in *Anacardiaceae*: mango and cashew experienced lineage-specific polyploidy, whereas pistachio and Rhus diversified through transposable element (TE)-driven SV. These SVs coincided with expansions and clustering of *WRKY* and *NLR* disease resistance genes, highlighting TE-mediated SVs as engines of immune diversification. Parallel findings have emerged in other plant species. In pepper, for example, retroduplication and TE bursts massively expanded NLR repertoires, accelerating pericentromeric evolution and strengthening immune capacity (Kim et al., 2021). Across the legume genus, pan-NLRome studies emphasized that SV-mediated birth–death cycles, recombination, and copy number changes are central to immune evolution (Wang et al., 2025). Tong et al. (2025) revealed that the expansion of the *bHLH* gene family in barley may be enriched in specific subfamilies, implying a potential link to species-specific environmental adaptation at the pangenome level. Together, these studies underscore a shared theme: pangenome analyses reveal how SVs, from whole-genome and local duplications to TE-mediated rearrangements, drive lineage-specific innovation in development and defense (Jia et al., 2023). Beyond evolutionary insights, these findings generate prioritized gene and allele catalogs that can be leveraged for crop improvement, illustrating the dual significance of pangenomics for both fundamental research and applied plant science.

## Broader advances in plant pangenomics and SV analysis

Findings from this Research Topic also align with those of recent large-scale plant pangenomic projects, underscoring the pervasive importance of SVs. In barley, a pangenome built from 76 high-quality assemblies and over 1,300 resequenced accessions uncovered thousands of PAVs and complex SVs distinguishing wild and cultivated accessions (Jayakodi et al., 2024). Many of these genetic variations were linked to agronomic traits: for example, the expansion of a starch-degrading enzyme family in elite malting barleys enhanced enzyme activity during grain malting, while the deletion of a regulatory enhancer altered awn morphology. These cases demonstrate how diversity at structurally complex loci has enabled barley to adapt to agricultural environments and specialized uses. In rice, Guo et al. (2025) assembled 145 genomes (129 wild *O. rufipogon* and 16 cultivated *O. sativa*), identifying 3.87 Gb of novel sequences absent from the Nipponbare reference and a total of 69,531 genes, ~14,000 of which were specific to wild populations. Wild rice harbors far greater diversity of resistance genes than domesticated rice, emphasizing how domestication bottlenecks narrowed SV-driven resilience. Tens of thousands of SVs, including PAVs and inversions, also distinguished *indica* and *japonica* subspecies, shedding light on subpopulation adaptation and the domestication process. Together, these studies reinforce the core messages of this Research Topic that pangenomes reveal the hidden structural variation underlying trait diversity, and that integrating SV maps with functional validation transforms catalogs of variants into breeding markers. Across species, SVs frequently affect domesticated, stress resistance and immunity response traits, and leveraging wild germplasm through pangenomics provides valuable alleles lost during domestication.

## Conclusion

Taken together, the studies in this Research Topic highlight innovations in plant pangenomics with a focus on SVs. From single-species pangenomes identifying candidate genes for stress tolerance to cross-species comparisons of evolutionary history, the contributions show how SVs shape plant diversity and adaptation. Common themes include building comprehensive pangenome resources, characterizing SV impacts on gene content and function, and integrating genomic with transcriptomic and phenotypic data. Along with recent large-scale plant genomics studies, these works underscore that SVs are fundamental drivers of evolution and breeding potential, paving the way for pangenome-guided crop improvement and sustainable agriculture (Hu et al., 2025).
